# Early Changes of Neuromuscular Transmission in the SOD1(G93A) Mice Model of ALS Start Long before Motor Symptoms Onset

**DOI:** 10.1371/journal.pone.0073846

**Published:** 2013-09-05

**Authors:** Mariana C. Rocha, Paula A. Pousinha, Alexandra M. Correia, Ana M. Sebastião, Joaquim A. Ribeiro

**Affiliations:** 1 Institute of Pharmacology and Neurosciences, Faculty of Medicine, University of Lisbon, Lisbon, Portugal; 2 Unit of Neurosciences, Institute of Molecular Medicine, University of Lisbon, Lisbon, Portugal; 3 Museu Nacional de História Natural e da Ciência, University of Lisbon, Lisbon, Portugal; Inserm, France

## Abstract

Amyotrophic lateral sclerosis is characterized by a progressive degeneration of the corticospinal tract motor neurons. Growing evidence suggests that degeneration may begin at the distal axon proceeding in a dying-back pattern. It seemed therefore of interest to investigate synaptic transmission at the neuromuscular junction (NMJ) in pre- and symptomatic phases of the disease. Endplate potentials (EPPs), miniatures endplate potentials (MEPPs) and giant MEPPs (GMEPPs) were recorded from innervated diaphragm muscle fibers from 4–6 and 12-15 weeks-old SOD1(G93A) mice and non-transgenic aged-matched littermates (WT). In the pre-symptomatic phase, SOD1(G93A) mice exhibited a significant increase in the mean amplitude of EPPs together with an increase in the mean *quantal content* of EPPs, suggesting that more acetylcholine is being released into the synaptic cleft. SOD1(G93A) mice presented a higher frequency of GMEPPs, suggestive of intracellular Ca^2+^ deregulation in nerve terminals. The increase in the mean amplitude of MEPPs and the decreased mean rise-time of MEPPs in SOD1(G93A) mice point to post-synaptic related changes. In the symptomatic phase, electrophysiological data showed evidence for two NMJ groups in SOD1(G93A) mice: SOD1a and SOD1b. SOD1a group presented reduced mean amplitude of both EPPs and MEPPs. The mean rise-time of MEPPs was increased, when compared to WT and to SOD1b group, indicating impairments in the neuromuscular transmission. In contrast, the neuromuscular transmission of SOD1b group was not different from age-matched WT nor pre-symptomatic SOD1(G93A) mice, being somehow in between both groups. Altogether these results show that the neuromuscular transmission of SOD1(G93A) mice is enhanced in the pre-symptomatic phase. In the symptomatic phase our results are consistent with the hypothesis that the diaphragm of SOD1(G93A) mice is undergoing cycles of denervation/re-innervation supported by mixed neuromuscular junction populations. These early changes in the neuromuscular transmission of SOD1(G93A) mice suggest that the ALS associated events start long before symptoms onset.

## Introduction

Amyotrophic Lateral Sclerosis (ALS) is the most common adult-onset motor neuron disease characterized by the degeneration of motor neurons in the corticospinal tract. This progressive loss leads to widespread muscle weakness, atrophy and paralysis, and ultimately to death after the involvement of the respiratory muscles. Although most cases have no known cause, 10% are inherited with 20% of these familial cases being caused by a mutation in the copper/zinc superoxide dismutase gene (Cu/Zn, SOD1). Both familial and sporadic forms of ALS present similar pathological and clinical features suggesting a common pathogenesis [1,2].

In the past decade, several studies have been pointing to a dying-back hypothesis, with an initiation at the motor endplate followed by a retrograde degeneration [3–6]. While this hypothesis suggests the second motor neuron as the first structure of the nervous system to be affected by the disease, little is known about ALS related changes at that the neuromuscular junction level. Contradictory results were published regarding the neuromuscular transmission. Kim and colleagues [7] reported that mean *quantal content* of endplate potentials (EPPs) is increased in symptomatic SOD1(G93A) mice whereas both frequency and amplitude of miniature endplate potentials (MEPPs) are not modified. More recently, Naumenko and colleagues [8] showed that the mean frequency of MEPPs is dramatically reduced in symptomatic congenic SOD1(G93A) male and female mice, without marked differences neither in the amplitude of MEPPs nor in the amplitude and *quantal content* of EPPs. Regarding the pre-symptomatic phase of the disease, Souayah and colleagues [9] reported that pre-symptomatic SOD1(G93A) mice (6 weeks-old) present a reduced probability of successful neuromuscular transmission at high frequencies of stimulation (70 and 90 Hz).

To appraise the time-related changes at different disease time points we evaluated transmission in both pre-symptomatic and symptomatic phases of the disease in the SOD1(G93A) mice under similar experimental conditions. The results now reported show that the mice-diaphragm neuromuscular junction from SOD1(G93A) mice undergoes functional changes early before the symptomatic phase, which do not evolve in the same direction after appearance of motor symptoms.

## Methods

### Ethics statement

This study was carried out in accordance with the European Community guidelines (Directives 86/609/EU and 2010/63/EU, Recommendation 2007/526/CE, European Convention for the Protection of Vertebrate Animals used for Experimental or Other Scientific Purposes ETS 123/Appendix A) and Portuguese Laws on Animal Care (Decreto-Lei 129/92, Portaria 1005/92, Portaria 466/95, Decreto-Lei 197/96, Portaria 1131/97). The protocols used in this study were approved by the Portuguese National Authority (General Direction of Veterinary) and by the Ethics Committee of the Institute of Molecular Medicine.

### Animals

Transgenic males B6SJL-TgN(SOD1-G93A) 1Gur/J (Jackson Laboratory, No. 002726), carrying the human SOD1 gene with the G93A point mutation [10] and wild-type B6SJLF1/J females were purchased from Jackson Laboratory (USA) and a colony was established at the rodent facility of the Institute of Molecular Medicine. At time of weaning, littermates were identified through ear punching and separated in different cages according to their gender. The ear tissue extracted during the tagging procedure was then used to run a polymerase chain reaction (PCR) and genotype the progeny [11]. All animals were housed 4-5 animals/cage, under 12h light/12h dark cycle, and received food and water *ad libitum*.

Experiments were conducted in two age groups: 4-6 and 12-15 weeks-old animals, corresponding to the pre-symptomatic and symptomatic phases of the disease, respectively. Both males and females were used, being the proportion between them similar in all groups (Tables 1-4). Transgenic mice, hereafter referred to as SOD1(G93A) mice, were compared to non-transgenic mice, used as age-matched controls (WT).

**Table 1:** Motor profile of the pre-symptomatic and symptomatic SOD1(G93A) mice.

	4-6 weeks old		12-15 weeks old	
	WT	SOD1(G93A)	WT	SOD1(G93A)
n (mice)	16 (9M, 7F)	11 (5M, 6F)	10 (4M, 6F)	10 (6M, 4F)
Body weight (g)	19 ± 0.72	18 ± 0.95	24 ± 0.93	24 ± 0.89
LF at 5 rpm (s)	242 ± 13.4	262 ± 14.0	267 ± 12.3	246 ± 23.6
LF at10rpm (s)	257 ± 11.8	236 ± 21.8	263 ± 13.9	181 ± 31.3^#^

The values are mean±SEM. Results obtained from aged-matched SOD1(G93A) and WT mice were compared (#p<0.05, Mann-Whitney test). LF, latency-to-fall; rpm, rotations-per-minute

**Table 2:** The neuromuscular transmission of SOD1(G93A) mice in pre-symptomatic phase of the disease.

	4-6 weeks old	Pre-symptomatic
	WT	SOD1(G93A)
n (fiber, mice)	40, 18	40,13
Male	17, 7	31, 10
Female	23, 11	9, 3
Mean age (days)	38.5 ± 1.08	41.2 ± 1.05
RMP (mV)	-69.0 ± 1.74	-67.6 ± 1.80
EPPs amplitude (mV)	16.6 ± 1.48	24.3 ± 1.61^#^
EPPs amplitude _NOR_ (mV)	18.3 ± 1.64	26.8 ± 1.54^#^
*Quantal content*_NOR_	29.0 ± 1.89	38.1 ± 2.42^#^
MEPPs amplitude(mV)	0.55 ± 0.02	0.63 ± 0.02^#^
MEPPs rise-time (ms)	1.32 ± 0.07	1.11 ± 0.08^#^
MEPPs decay time (ms)	4.00 ± 0.15	3.67 ± 0.14
MEPPs frequency (s^-1^)	0.64 ± 0.07	0.59 ± 0.06
N^°^ of NMJs with GMEPPs	30	33
GMEPPs frequency (s^-1^)	0.23 ± 0.06	0.44 ± 0.08^#^
Fr(GMEPPs)/Fr(MEPPs)	2.27 ± 1.35	1.86 ± 0.45

The values are mean±SEM. Results obtained from SOD1(G93A) and age-matched WT mice were compared (#p<0.05 Mann-Whitney test). EPP, endplate potential; EPP amplitude _NOR_, endplate potential normalized to a resting membrane potential(RPM) of -75mV; MEPP, miniature endplate potential; Quantal content_NOR,_ normalized quantal content of EPPs (meanEPP_NOR_/meanMEPP_NOR_); Fr(MEPPs), MEPPs frequency; Fr (GMEPPs), GMEPPs frequency; NMJ, neuromuscular junction

**Table 3:** The neuromuscular transmission of SOD1(G93A) mice in symptomatic phase of the disease.

	12-15 weeks old	Symptomatic
	WT	SOD1(G93A)
n (fiber, mice)	30, 11	39, 11
Male	17, 5	21, 5
Female	13, 6	18, 6
Mean age (days)	98.2 ± 0.78	99.6 ± 0.46
RMP (mV)	-64.8 ± 2.50	-65.8 ± 1.69
EPPs amplitude (mV)	21.0 ± 2.45	18.3 ± 1.88
EPPs amplitude _NOR_ (mV)	23.6 ± 2.21	18.3 ± 1.88
*Quantal content*_NOR_	41.3 ± 3.79	35.8 ± 2.74
MEPPs amplitude (mV)	0.50 ± 0.03	0.49 ± 0.03
MEPPs rise-time (ms)	0.98 ± 0.07	1.15 ± 0.07
MEPPs decay-time (ms)	3.12 ± 0.14	3.21 ± 0.13
MEPPs frequency (s^-1^)	0.76 ± 0.11	0.80 ± 0.11
N^°^ of NMJs with GMEPPs	16	20
GMEPPs frequency (s^-1^)	0.44 ± 0.12	0.32 ± 0.10
Fr(GMEPPs)/Fr(MEPPs)	1.58 ± 0.49	1.21 ± 0.68

The values are mean±SEM. Results obtained from age-matched SOD1(G93A) and WT mice were compared. EPP, endplate potential; EPP amplitude _NOR_, endplate potential normalized to a resting membrane potential(RPM) of -75mV; MEPP, miniature endplate potential; Quantal content_NOR,_ normalized quantal content of EPPs(meanEPP_NOR_/meanMEPP_NOR_); Fr(MEPPs), MEPPs frequency; Fr (GMEPPs), GMEPPs frequency; NMJ, neuromuscular junction

### Phase of the disease assessment

To establish the age groups correspondent to the pre-symptomatic and symptomatic stage of the disease, mice were tested on a Rotarod. A training period comprising two stages was performed in order to minimize environmental variability. First animals were habituated to handling - initiated four days before the testing day. Then animals were habituated to the Rotarod apparatus and were taught the task itself. For that, mice were placed on the rod at the lowest rotation speed (4 rpm) where they had to maintain for at least 120s, as described in [12]. In the testing day, mice were sequentially assessed at 5 and 10 rpm, for a maximum of 300s each speed (as similarly done by [13]). The latency-to-fall (LF), which corresponds to the time that mice spent on the rotating rod, was recorded. 3 trials were performed per speed with 5 min rest between each trial.

### Electrophysiological recordings

Mice were sacrificed by decapitation under halothane anaesthesia. Hemi-diaphragm muscle with the attached phrenic-nerve was isolated and mounted on a 3ml perspex chamber. Tissue was perfused continuously with a gassed physiological saline solution (containing (mM): NaCl 117; KCl 5; NaHCO_3_ 25; NaH_2_PO_4_ 1,2; glucose 11; CaCl_2_ 2,5; MgCl_2_ 1,2; 95% O_2_ and 5% CO_2_) kept at room temperature (22-25^°^C). Perfusion was maintained at a rate of 3ml/min *via* a roller pump, and bath volume was kept constant by suction.

Intracellular recordings were performed in the conventional way [14–16]. Glass microelectrodes (10-20 MΩ) filled with 3M KCl were inserted within muscle fibers at the motor endplate and the reference electrode was an Ag–AgCl pellet placed in the bath. Muscle contraction was prevented by treating preparations with 2µM μ-conotoxin GIIIB [17] for 45 to 60min, as done previously by [18]. The toxin selectively blocks voltage dependent sodium channels in the muscle fiber thereby preventing muscle action potential generation. In this experimental condition, neuromuscular transmission occurs at normal *quantal content* without interference of nicotinic acetylcholine receptors. The phrenic-nerve was stimulated supramaximally at 0.5 Hz with a constant current of 20 µs duration. The evoked activity (EPPs) at each neuromuscular junction was measured during intervals of 10 minutes. MEPPs were acquired in gap free mode during 100s. The resting membrane potential of the muscle fiber was monitored through the entire experiment. Signals were amplified, digitalized and stored on a computer with the Clampex program (pClamp10 Axon Instruments, Foster City, USA), for offline analysis of data.

Neuromuscular junctions considered for analysis had a stable resting membrane potential ranging from -60mV to -80mV. EPP amplitudes were normalized to a membrane potential of -75 mV using the following equation V_NOR_ = [V_OBS_ x (-75)]/RMP, where V_NOR_ indicates the corrected amplitude, V_OBS_ the recorded amplitude and RMP the recorded resting membrane potential [19–21]. The mean amplitude of EPPs was calculated for each neuromuscular junction by averaging normalized EPP amplitudes of 60 consecutive sweeps. The *quantal content* of EPPs, was estimated through the ratio between the normalized average of EPPs amplitude and the normalized average of MEPPs amplitude, both acquired during the same period. The threshold for MEPPs detection was set at levels between 0.2 mV and 1mV of amplitude. This maximum amplitude was established in order to exclude giant MEPPs (GMEPPs). The mean amplitude of MEPPs was calculated by averaging the amplitude of 100 consecutive MEPPs. The same procedure was done to assess the mean rise-time of MEPPs and mean decay-time of MEPPs. The mean frequency of MEPPs was measured by counting the number of events acquired during 100s. The distribution of MEPPs amplitudes was approximated to a Gaussian function or a Sum of two Gaussian function and the goodness of fit was evaluated by the square correlation coefficient R^2^. The minimum amplitude for GMEPPs detection was set at 1mV. The mean frequency of GMEPPs was obtained by counting the number of these events acquired during the 100s. To evaluate the probability of GMEPPs occurrence between groups, the mean ratio between frequency of GMEPPs and frequency of MEPPs was calculated for each fiber exhibiting GMEPPs.

### Statistical analysis

All data are presented as mean ± standard error mean (SEM) in each group, with n corresponding to the number of animals used or the number of neuromuscular junctions sampled, as indicated in each figure or table.

Statistical significance of differences between means was determined through GraphPad software. The parametric Student’s t test was used whenever both groups presented a normal distribution and equality of variances. Normality was tested through Shapiro-Wilk test (S–W), which is more appropriate for small samples (n<30), and homogeneity of variances tested through F test. Alternatively, the non-parametric Mann–Whitney U-test (M-W) was applied when one of the groups was non-normally distributed and Student’s t test with Welsh correction when the equality of variances condition was not met (F test p<0.05).

**Table 4:** The neuromuscular transmission of the two populations of neuromuscular junctions detected in symptomatic SOD1(G93A) mice.

	12-15 weeks old	Symptomatic	
	WT	SOD1a	SOD1b
n (fiber, mice)	30, 11	19, 8	20, 10
Males	17, 5	11, 4	10, 5
Females	13, 6	8, 4	10,5
Age range (days)	89-108	94-107	94-102
RMP (mV)	-64.8 ± 2.50	-64.4 ± 2.05	-67.1 ± 2.67
EPPs amplitude (mV)	21.0 ± 2.45	11.4 ± 2.00 ^#WT^	24.9 ± 2.33 ^#SOD1a^
EPPs amplitude _NOR_ (mV)	23.6 ± 2.21	12.8 ± 2.06 ^#WT^	28.1 ± 2.41
*Quantal content*_NOR_	41.3 ± 3.79	32.7 ± 4.40	38.8 ± 3.30
MEPPs amplitude (mV)	0.50 ± 0.03	0.32 ± 0.02 ^#WT^	0.64 ± 0.02 ^#WT #SOD1a^
MEPPs rise-time (ms)	0.98 ± 0.07	1.39 ± 0.12 ^#WT^	0.93 ± 0.06 ^#SOD1a^
MEPPs decay-time(ms)	3.12 ± 0.14	3.12 ± 0.17	3.28 ± 0.20
MEPPs frequency (s^-1^)	0.76 ± 0.11	0.61 ± 0.11	1.26 ± 0.33
N^°^ of NMJs with GMEPPs	16	3	17
GMEPPs frequency (s^-1^)	0.44 ± 0.12	0.07 ± 0.03 **^+^** ^WT^	0.38 ± 0.12 **^+^** ^SOD1a^
Fr(GMEPPs)/Fr(MEPPs)	1.58 ± 0.49	0.11 ± 0.07 **^+^** ^WT^	1.40 ± 0.80

The values are mean±SEM. Results from SOD1a and SOD1b NMJs were compared with age-matched WT NMJs (indicated with WT symbol next to the statistical significance symbol) and with each other (indicated with SOD1a symbol next to the statistical significance) (#p<0.05, Mann-Whitney test and +p<0.05, Student test with Welsh correction). EPP, endplate potential; EPP amplitude _NOR_, endplate potential normalized to a resting membrane potential(RPM) of -75mV; MEPP, miniature endplate potential; Quantal content_NOR,_ normalized quantal content of EPPs (meanEPP_NOR_/meanMEPP_NOR_); Fr(MEPPs), MEPPs frequency; Fr (GMEPPs), GMEPPs frequency; NMJ, neuromuscular junction

## Results

### Motor phenotype of pre-symptomatic and symptomatic SOD1(G93A)

As shown in Table 1 the mean latency-to-fall of 4-6 weeks old SOD1(G93A) mice did not differ from WT mice neither at 5 rpm (262 ± 14.0s, n=11, and 242±13.4, n=16, respectively, p>0.05, Mann Whitney test), nor at 10 rpm (236±21.8s, n=11, and 257±11.8s, n=16, respectively, p>0.05, Mann Whitney test). Thus, we established the age of 4-6 weeks old as the pre-symptomatic SOD1(G93A) group. In contrast, the performance of 12-15 weeks old SOD1(G93A) mice on the Rotarod task was significantly decreased when compared to WT mice. The mean latency-to-fall, not different between groups at 5 rpm (267±12.3s, n=10, in WT and 246±23.6s n=10, in SOD1(G93A) groups, p>0.05, Mann Whitney test), became significantly reduced by 31% at 10 rpm (263±13.9s, n=10, in WT and 181±31.3s, n=10, in SOD1(G93A) groups, p<0.05, Mann Whitney test). We therefore established this group as the symptomatic SOD1(G93A) mice.

### Pre-symptomatic phase of ALS

**Figure 1:**
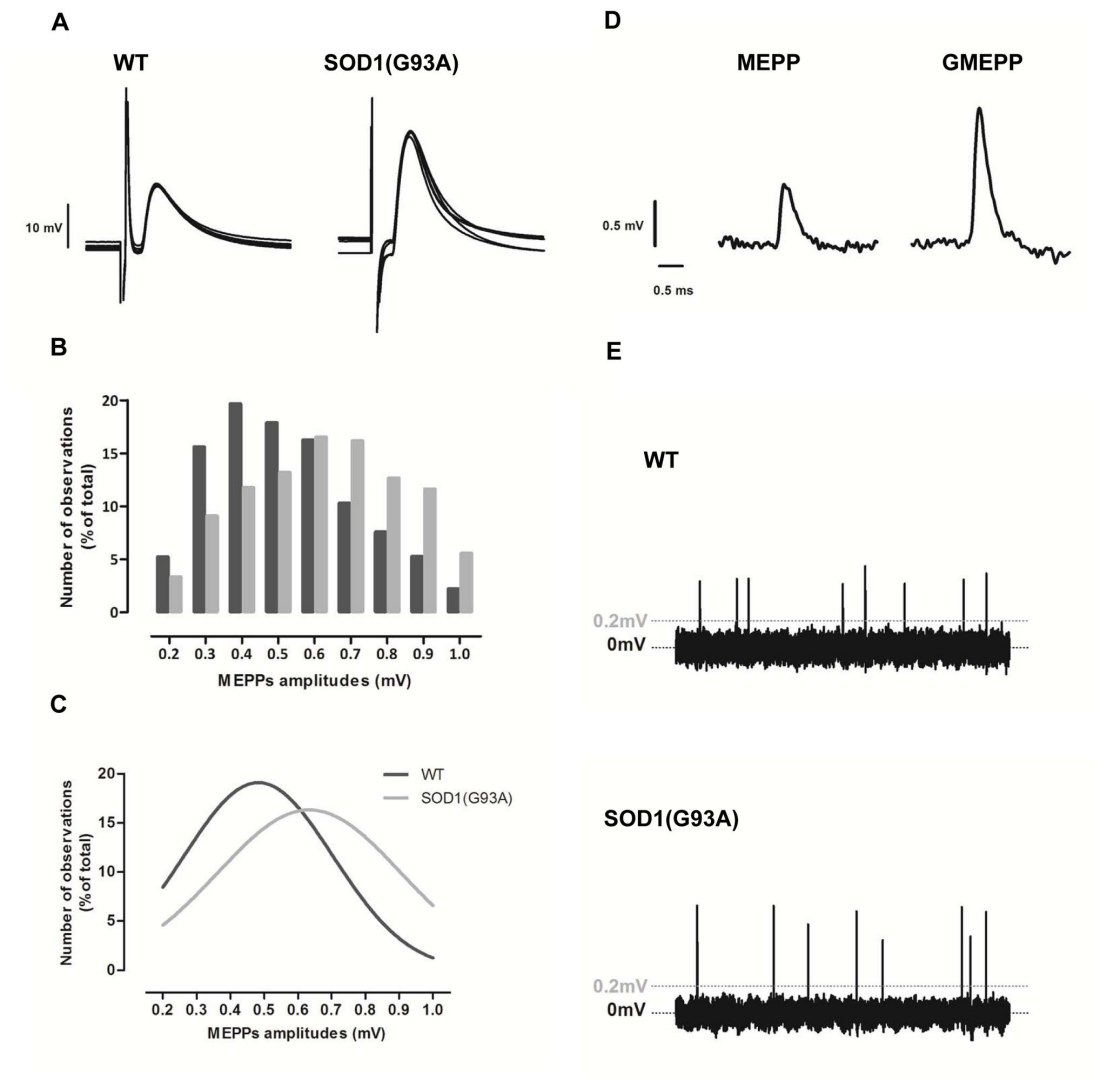
The neuromuscular transmission of the SOD1(G93A) mice in the pre-symptomatic phase of the disease. A) Illustrates raw recordings of 5 EPPs from 4–6 week-old WT and pre-symptomatic SOD1(G93A) mice. B) Shows the frequency histogram of MEPPs amplitudes, which pools the amplitude of all MEPPs recorded at WT (2785 MEPPs) and SOD1(G93A) (2270 MEPPs) fibers. WT – Black bars; SOD1(G93A) – Gray bars. C) Presents the nonlinear regression applied to WT and SOD1(G93A) distributions. As illustrated, both distributions were best fitted with a Gaussian function. In D) are shown examples of a MEPP (<1mV) and a GMEPP (>1mV) and in E) examples of spontaneous events recorded in gap free mode across 10s in WT and SOD1(G93A) neuromuscular junctions.

The evoked activity of pre-symptomatic SOD1(G93A) mice is presented in Figure 1.A and further quantified in table 2. Pre-symptomatic SOD1(G93A) mice presented a mean amplitude of EPPs significantly higher than WT mice (16.6±1.48mV, n=40, in WT group and 24.3±1.61mV, n=40, in SOD1(G93A) group, p<0.05, Mann Whitney test). This difference was maintained after the normalization of muscle fiber resting membrane potential to -75mV (18.3±1.64mV, n=40, in WT and 26.8±1.54mV, n=40, in SOD1(G93A) mice, p<0.05, Mann Whitney test). Similarly, the mean *quantal content* of SOD1(G93A) mice was significantly enhanced by approximately 31% in relation to WT mice (29.0±1.89, n=40, in WT and 38.1±2.42, n=40, in SOD1(G93A) mice, p<0.05, Mann Whitney test). The mean resting membrane potential did not differ between WT and SOD1(G93A) mice (p>0.05, Mann Whitney test) (Table 2).

As also shown in table 2 and Figure 1, the mean amplitude of MEPPs was significantly higher in SOD1(G93A) mice by approximately 15% when compared to WT mice (0.55±0.02mV, n=40, in WT and 0.63±0.02mV, n=40, in SOD1(G93A) mice, p<0.05, Mann Whitney test). This difference is more outstanding when looking at the distribution of MEPPs amplitudes (Figure 1.B) and to the Gaussian functions that shape the scatter of data (being the values of R^2^ equal to 0.93 and 0.88 for WT and SOD1(G93A) curves respectively) (Figure 1.C). The distribution of data from SOD1(G93A) presents a pronounced skewing to the right, highlighting the shift to larger amplitudes of MEPPs in this group. The frequency of MEPPs in pre-symptomatic SOD1(G93A) mice (0.59±0.06s^-1^, n=40) did not differ from WT mice (0.64±0.07s^-1^, n=40) (p>0.05, Mann Whitney test). As illustrated in Figure 1.D,E and Table 2, the mean frequency of GMEPPs was significantly increased in SOD1(G93A) mice (0.44±0.08 s^-1^, n=33) in relation to the WT mice (0.23±0.06 s^-1^, n=30) (p<0.05, Mann Whitney test). Moreover, the mean ratio between the frequency of GMEPPs and the frequency of MEPPs (calculated for each fiber exhibiting GMEPPs) was increased in SOD1(G93A) group (2.27±1.35, n=30) by 82% when compared with WT group (1.86±0.45, n=33) (p>0.05, Mann Whitney test) indicating that the occurrence of a GMEEPs is proportionally higher than a MEPP.

As shown in table 2, the mean rise-time of MEPPs in SOD1(G93A) mice was significantly decreased (1.32±0.07ms, n=40, in WT and 1.11±0.08ms, n=40, in SOD1(G93A) mice, p<0.05, Mann Whitney test) in relation to WT mice. No differences on the mean decay-time of MEPPs between SOD1(G93A) (3.67±0.14ms, n=40) and WT (4.00±0.15ms, n=40) (p>0.05, Mann Whitney test) mice were found.

### Symptomatic phase of ALS

**Figure 2:**
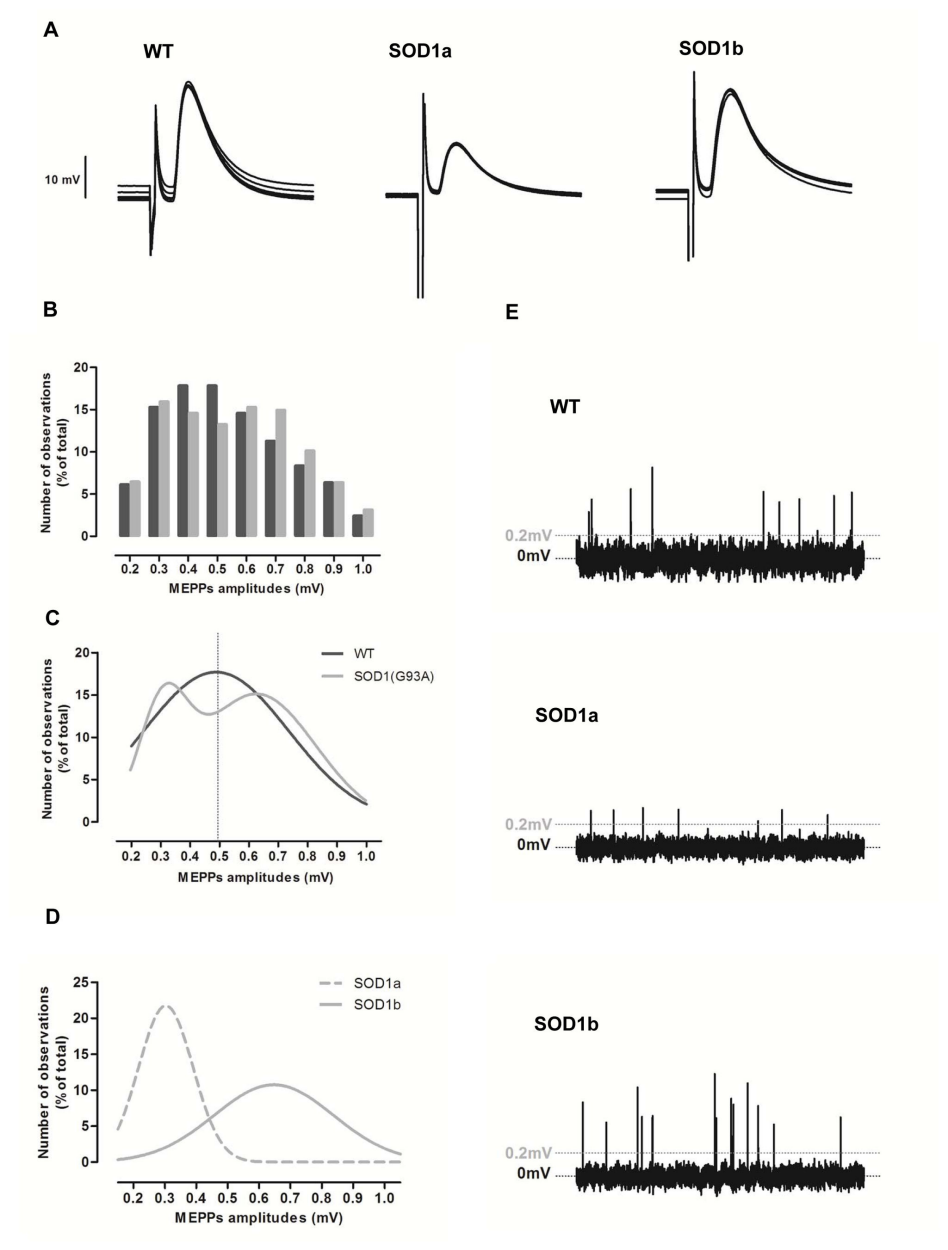
The neuromuscular transmission of the SOD1(G93A) mice in the symptomatic phase of the disease. A) Illustrates raw recordings of 5 EPPs from adult WT mice and the two groups of symptomatic SOD1(G93A) neuromuscular junctions, SOD1a and SOD1b. B) Shows the frequency histogram of MEPPs amplitudes, that pools the amplitude of all MEPPs recorded at WT (2288 events) and SOD1(G93A) (3674 events) fibers. WT – Black bars; SOD1(G93A) – Gray bars. C) Shows the nonlinear regressions that best shape WT (Gaussian function) and SOD1(G93A) (sum of two Gaussians) data As illustrated, SOD1(G93A) data follows a bimodal distribution with two peak amplitudes, pointing to the existence of 2 groups of neuromuscular junctions. D) After categorizing the two groups, the distributions of MEPPs amplitudes from SOD1a and SOD1b groups were drawn and best-fitted with Gaussian functions. As it is visible, the peak amplitude from both distributions matches the two peak amplitudes from the bimodal curve, validating the grouping. E) Shows examples of spontaneous events recorded in gap free mode across 10s in WT, SOD1a and SOD1b neuromuscular junctions.

The evoked response of symptomatic SOD1(G93A) mice is illustrated in Figure 2.A and quantifications shown in detail in Table 3. The mean amplitude of EPPs (normalized to a resting membrane potential of -75mV and non-normalized) and the mean *quantal content* of EPPs in SOD1(G93A) did not significantly differ from WT (p>0.05, Mann Whitney test). The mean resting membrane potential was also maintained between groups (p>0.05, Student t test).

As shown in table 3 and illustrated in Figure 2.B, the mean amplitude of MEPPs did not vary between WT and SOD1(G93A) mice (0.50±0.03mV, n=30, and 0.49±0.03mV, n=39, respectively, p>0.05, Mann Whitney test) indicating no apparent changes in the post-synaptic cell. We noticed, however, that the distribution of SOD1(G93A) MEPPs amplitudes did not follow a Gaussian distribution. Instead, a sum of two Gaussian functions best fitted the distribution of SOD1(G93A) data, as shown in Figure 2.C (first peak at 0.31±0.01 mV and second peak at 0.63±0.01mV, R^2^=0.996), raising the possibility of two populations of neuromuscular junctions. To investigate this, we established the mean amplitude of SOD1(G93A) MEPPs (0.49mV) as a limit to categorize neuromuscular junctions into two groups – group A with a mean amplitude of MEPPs lower than 0.49mV and group B equal or higher than 0.49 – which will be referred to as SOD1a and SOD1b, respectively, in the following text As illustrated in Figure 2.D, the distributions of MEPPs amplitudes from both groups of neuromuscular junctions were best fitted with Gaussian functions. The peak amplitudes of each group (Group SOD1a peak amplitude at 0.30±0.01 mV, R^2^=0.90, and Group SOD1b peak amplitude at 0.65±0.01mV, R^2^=0.97) matched the two peak amplitudes from the bimodal curve, an indicator of proper grouping.

Regarding the frequency of spontaneous events, the mean frequency of MEPPs did not differ between WT and symptomatic SOD1(G93A) mice (p>0.05, Mann Whitney test) (Table 3, Figure 2.E). The mean frequency of GMEPPs was reduced by approximately 27% in SOD1(G93A) mice (0.32±0.10s^-1^, n=20) as compared to WT mice (0.44±0.12s^-1^, n=16) (p>0.05, Mann Whitney test). Accordingly, the proportion of GMEPPs (ratio between the frequency of GMEPPs and the frequency of MEPPs) was decreased by 23% in SOD1(G93A) group (1.21±0.68, n=20) when compared to WT mice (1.58±0.49, n=16) (p>0.05, Mann Whitney test).

Once the two groups of symptomatic SOD1(G93A) neuromuscular junctions were established, evoked and spontaneous activity were re-analyzed in order to delineate the profile of each group, as shown in table 4. The SOD1a group presented a mean amplitude of EPP significantly reduced (21.0±2.45mV, n=30, in WT and 11.4±2.00mV, n=19, in SOD1a groups, p<0.05, Mann Whitney test). Also, the *quantal content* of EPPs was slightly decreased in relation to WT (41.3±3.79, n=30, in WT and 32.7±4.40, n=19, in SOD1a, p>0.05, Mann Whitney test), although not statistically significant. The mean frequency of MEPPs was approximately the same in both SOD1a and WT groups (0.61±0.11s^-1^, n=19, and 0.76±0.11s^-1^, n=30, respectively, p>0.05, Mann Whitney test). However, GMEPPs were more rare in SOD1a (3 out of 19 neuromuscular junctions, 16%) than in WT (16 out of 30 neuromuscular junctions, 53%). In addition, SOD1a exhibited a mean frequency of GMEPPs significantly reduced when compared to WT (0.44±0.12s^-1^, n=16 in WT and 0.07±0.03 s^-1^, n=3, in SOD1a groups, p<0.05, Student t test with Welsh correction) as well as a lower mean ratio between the frequency of GMEPPs and the frequency of MEPPs (1.58±0.49, n=16, in WT and 0.11±0.07, n=3, in SOD1a groups, p<0.05, Student t test with Welsh correction). SOD1a presented a lower mean amplitude of MEPPs (0.50±0.03mV, n=30 in WT and 0.32±0.02, n=19, in SOD1a groups, p<0.05, Mann Whitney test), a higher mean rise-time of MEPPs (0.98±0.07ms, n=30, in WT and 1.39±0.12ms, n=19, in SOD1a groups, p<0.05, Mann Whitney test), and no differences in the mean decay-time of MEPPs (3.12±0.14ms, n=30, in WT and 3.12±0.17ms, n=19, in SOD1a groups, p>0.05, Mann Whitney test). In contrast, most of the parameters investigated in SOD1b group did not show significant differences (p>0.05, Mann Whitney test or Student t test, depending on the normality of the parameter analyzed; Table 4) when compared to the WT group, the exception being the mean amplitude of MEPPs, which was significantly increased as compared with the WT group (0.50±0.03mV, n=30 in WT and 0.64±0.02, n=20, in SOD1b groups, p<0.05, Mann Whitney test).

### Comparison between phases

To investigate how the neuromuscular transmission evolved from pre-symptomatic to symptomatic SOD1(G93A) mice we further compared both phases of the disease (Table 5) taking into account SOD1a and SOD1b neuromuscular junctions. As shown, SOD1a neuromuscular junctions revealed a marked reduction in the mean amplitude of EPPs (24.3±1.61mV, n=40, in pre-symptomatic SOD1(G93A) and 11.4±2.00mV, n=19, in SOD1a, p<0.05, Mann Whitney test) and no significant variation of the mean *quantal content* of EPPs (p>0.05, Mann Whitney test), when compared to pre-symptomatic SOD1(G93A) mice.

**Table 5:** Evolution of the neuromuscular transmission from pre-symptomatic to symptomatic phases of the disease.

	Pre-symptomatic	Symptomatic	
	SOD1(G93A)	SOD1a	SOD1b
n (fiber, mice)	40, 18	19, 8	20, 10
Males	17, 7	11, 4	10, 5
Females	23, 11	8, 4	10,5
Age range (days)	89-108	94-107	94-102
RMP (mV)	-67.6 ± 1.80	-64.4 ± 2.05	-67.1 ± 2.67
EPPs amplitude (mV)	24.3 ± 1.61	11.4 ± 2.00^#^	24.9 ± 2.33
EPPs amplitude _NOR_ (mV)	26.8 ± 1.54	12.8 ± 2.06^#^	28.1 ± 2.41
*Quantal content*_NOR_	38.1 ± 2.42	32.7 ± 4.40	38.8 ± 3.30
MEPPs amplitude (mV)	0.63 ± 0.02	0.32 ± 0.02^#^	0.64 ± 0.02
MEPPs rise-time (ms)	1.11 ± 0.08	1.39 ± 0.12^#^	0.93 ± 0.06
MEPPs decay-time (ms)	3.67 ± 0.14	3.12 ± 0.17*	3.28 ± 0.20
MEPPs frequency (s^-1^)	0.59 ± 0.06	0.61 ± 0.11	1.26 ± 0.33
N^°^ of NMJs with GMEPPs	33	3	17
GMEPPs frequency (s^-1^)	0.44 ± 0.08	0.07 ± 0.03^+^	0.38 ± 0.12
Fr(GMEPPs)/Fr(MEPPs)	1.86 ± 0.45	0.11 ± 0.07^+^	1.40 ± 0.80

The values are mean±SEM. Results from symptomatic SOD1a and SOD1b NMJs were both compared with pre-symptomatic SOD1(G93A) NMJs (*p<0.05, Student test; #p<0.05, Mann-Whitney test and +p<0.05, Student test with Welsh correction). EPP, endplate potential; EPP amplitude _NOR_, endplate potential normalized to a resting membrane potential (RPM) of - 75mV; MEPP, miniature endplate potential; *Quantalcontent*_NOR,_ normalized *quantal content* of EPPs(meanEPP_NOR_/meanMEPP_NOR_); Fr(MEPPs), MEPPs frequency; Fr(GMEPPs), GMEPPs frequency; NMJ, neuromuscular junction.

Regarding spontaneous activity, the SOD1a group presented a decrease in the mean amplitude of MEPPs (0.63±0.02mV, n=40, in pre-symptomatic SOD1(G93A) and 0.32±0.02, n=19, in SOD1a, p<0.05, Mann Whitney test) as well as increased rise-time of MEPPs (1.11±0.08ms, n=40, in WT and 1.39±0.12, n=19, in SOD1a, p<0.05, Mann Whitney test) and lower decay-time of MEPPs (3.67±0.14ms, n=40, in WT and 3.12±0.17ms, n=19, in SOD1a, p<0.05, Student t test). The mean frequency of MEPPs remained unchanged but both mean frequency of GMEPPs and mean ratio (between the frequency of GMEPPs and frequency of MEPPs) were significantly reduced (p<0.05, Student t test with Welsh correction). Interestingly, in any of the quantified parameters did the SOD1b neuromuscular junctions differ in a statistically significant way (p<0.05, Mann Whitney test or Student t test depending on the normality of the parameter analyzed, Table 5) from the pre-symptomatic SOD1(G93A).

Functional differences between symptomatic SOD1(G93A) males and females have been reported [8]. However, the two identified populations of neuromuscular junctions (SOD1a and SOD1b) cannot be attributed to gender differences since SOD1a and SOD1b neuromuscular junctions were observed in either males (11 SOD1a and 10 SOD1b) or females (8 SOD1a and 10 SOD1b) and the proportion of females and males in SOD1a and SOD1b groups was similar (Tables 4-5). Furthermore, both SOD1a and SOD1b endplates were identified in the same hemi-diaphragm. Indeed, 7 of the 11 symptomatic SOD1(G93A) mice presented both groups SOD1a and SOD1b neuromuscular junctions, being these 3 females and 4 males. The age ranges in both SOD1a and SOD1b groups were also fully overlapping (Tables 4 and 5).

The negative trend seen in SOD1(G93A) between different time points contrasts with the positive trend from WT across age. As shown in Table 6, adult WT showed an increased mean *quantal content* (29.0±1.89, n=40, in young WT and 41.3±3.79, n=30, in adult WT, p<0.05, Mann-Whitney test), in accordance with what has been previously described [22] Regarding spontaneous activity, the mean amplitude of MEPPs was maintained between ages while the mean rise-time (1.32±0.07, n=40, in young WT and 0.98±0.07, n=30, in adult WT, p<0.05, Mann-Whitney test) and mean decay-time (4.00±0.15, n=40, in young WT and 3.12±0.14, n=30, in adult WT, p<0.05, Mann-Whitney test) of MEPPs appeared significantly reduced in adult WT (Table 6), compatible with age-related post-synaptic membrane maturation [23]. These changes highlight the need for age-matched controls use whenever comparing alterations in symptomatic and pre-symptomatic ALS mice models.

**Table 6:** Evolution of WT neuromuscular transmission with age.

	4-6 weeks old	12-15 weeks old
	WT	WT
n (fiber, mice)	40, 18	30, 11
Male	17, 7	17, 5
Female	23, 11	13, 6
Mean age (days)	38.5 ± 1.08	98.2 ± 0.78
RMP (mV)	-69.0 ± 1.74	-64.8 ± 2.50
EPPs amplitude (mV)	16.6 ± 1.48	21.0 ± 2.45
EPPs amplitude _NOR_ (mV)	18.3 ± 1.64	23.6 ± 2.21
*Quantal content*_NOR_	29.0 ± 1.89	41.3 ± 3.79^#^
MEPPs amplitude (mV)	0.55 ± 0.02	0.50 ± 0.03
MEPPs rise-time (ms)	1.32 ± 0.07	0.98 ± 0.07^#^
MEPPs decay-time (ms)	4.00 ± 0.15	3.12 ± 0.14^#^
MEPPs frequency (s^-1^)	0.64 ± 0.07	0.76 ± 0.11
N^°^ of NMJs with GMEPPs	30	16
GMEPPs frequency (s^-1^)	0.23 ± 0.06	0.44 ± 0.12
Fr(GMEPPs)/Fr(MEPPs)	2.27 ± 1.35	1.58 ± 0.49

The values are mean±SEM. Results obtained from pre-symptomatic and symptomatic WT mice were compared (#p<0.05, Mann-Whitney test). EPP, endplate potential; EPP amplitude _NOR_, endplate potential normalized to a resting membrane potential(RPM) of -75mV; MEPP, miniature endplate potential; Quantal content_NOR,_ normalized quantal content of EPPs(meanEPP_NOR_/meanMEPP_NOR_); Fr(MEPPs), MEPPs frequency; Fr (GMEPPs), GMEPPs frequency; NMJ, neuromuscular junction

## Discussion

The results reported in this work show that the neuromuscular transmission of SOD1(G93A) mice presents changes along the progression of ALS symptoms. In the pre-symptomatic phase, the synaptic transmission at the phrenic-nerve diaphragm neuromuscular junction was enhanced. In the symptomatic phase two groups of neuromuscular junctions were detected: one group maintained the same profile as those from pre-symptomatic mice whereas the second group exhibited a compromised neuromuscular transmission.

Pre-symptomatic SOD1(G93A) mice presented an enhanced mean amplitude and *quantal content* of EPPs when compared with young controls, suggesting that more acetylcholine is being released to the synaptic cleft. These results point to an early maturation of SOD1(G93A) neuromuscular junction since pre-symptomatic SOD1(G93A) and the adult wild-type mice do not present differences in the evoked activity. This could be a compensatory response for an eventual early denervation. Furthermore, the differences observed in the evoked activity are due to both pre- and post-synaptic mechanisms since the mean frequency of GMEPPs (associated with changes in the nerve terminal) and the mean amplitude of MEPPs (associated with changes in the post-synaptic cell) were also enhanced.

It is known that GMEPPs are generated through a “constitutive secretion” of acetylcholine [24]. They are insensitive to nerve-terminal depolarization and extracellular Ca^2+^ [25,26] and are triggered by Ca^2+^ release from intracellular stores or following an impaired processing of recycled vesicles [27]. Consistent with this, symptomatic SOD1(G93A) mice have (1) a defective Ca^2+^ homeostasis [28], (2) present dysfunctional mitochondria in motor neurons [29], which have reduced ability to uptake efficiently cytoplasmic Ca^2+^ [30–32] and induce Ca^2+^ release from endoplasmatic reticulum stores in muscle fibers [33]. Also (3), IgG from ALS patients were reported to interact with nerve terminals activating IP_3_ receptors [34]. Together, these events may cause an increase in cytoplasmic Ca^2+^ levels thereby increasing the spontaneous synchronized release of acetylcholine. GMEPPs have also been reported to be enhanced under pathological conditions like paralysis [35], nerve terminal sprouting and synapse remodeling [36], nerve terminals in degeneration [37] and in several motor endplate diseases [38]. Thus, the results described here suggest that diaphragm muscle from SOD1(G93A) mice already presents morphological changes in the pre-symptomatic phase. Consistent with this, Fischer and collaborators [39] performed a spatiotemporal analysis of disease progression in SOD1(G93A) mice and observed that 40% of end plates were denervated at day 47. Here we show functional evidence that the molecular mechanism and morphological consequence related to denervation might start even earlier.

The enhancement of the mean amplitude of MEPPs in the pre-symptomatic SOD1(G93A) mice could be related to (1) an increased quantal package of transmitter through larger synaptic vesicles, (2) a decreased acetylcholinesterase activity, (3) a higher sensitivity of the muscle fiber to acetylcholine (through a higher affinity of nicotinic acetylcholine receptors to their ligand and/or higher nicotinic acetylcholine receptors expression) or (4) changes in fiber type composition. To our knowledge there are no studies addressing acetylcholine content in single vesicles in this model of ALS. However, pre-symptomatic SOD1(G93A) mice present lower levels of choline acetyltransferase at nerve terminals, compromising vesicular release of acetylcholine [40]. Decreased acetylcholinesterase activity seems also unlikely since the mean decay-time of MEPPs recorded in SOD1(G93A) mice was similar to the wild-type mice, indicating that acetylcholine hydrolysis is not changed. Acetylcholine receptors in SOD1(G93A) mice remain clustered at neuromuscular junction throughout 5-20 weeks of age [41], therefore the enhanced mean amplitude of MEPPs may not be related to changes in the expression/affinity of nicotinic acetylcholine receptors at the motor endplate. The presently reported faster rise-time of MEPPs in SOD1(G93A) mice suggest that pre-symptomatic SOD1(G93A) motor endplates might have a higher permeability to cations than wild-type mice. Changes in fiber-type composition (occurring in other motor neuron diseases such as severe spinal muscular atrophy [42]) may also occur. Indeed, previous studies have shown that large motor neurons innervating IIB and IID/X muscle fibers have higher vulnerability and degenerate prematurely [4,5]. Also, pre-symptomatic SOD1(G93A) mice present a significant proportion of atrophic fast-twitch fibers (by day 60) [43]. Since the mean amplitude of MEPPs is known to be inversely related to muscle fiber diameter [44], an increase in type I or IIA muscle fibers (which have a lower diameter) and/or an increased atrophy of type IIB and IID/X muscle fibers could explain the differences in MEPPs amplitudes observed in the pre-symptomatic phase of the disease.

In symptomatic SOD1(G93A) mice we could find two groups of neuromuscular junctions: one (group SOD1b) maintained the enhanced neuromuscular transmission observed in pre-symptomatic phase and the other (group SOD1a) exhibited impaired neuromuscular transmission, when compared with adult controls. Gender differences (see 8) could not account for these observations, since 4 males and 3 females symptomatic mice presented both SOD1a and SOD1b neuromuscular junctions. It has been reported [8] that the mean amplitude of EPPs, the *quantal content* of EPPs and the mean amplitude of MEPPs from symptomatic congenic SOD1(G93A) mice is not different from wild-type mice. Accordingly, we also observed no differences between symptomatic SOD1(G93A) mice and wild-type mice when considering the average of all neuromuscular junctions in which the recordings were performed (Table 3). However, as we now show, the distribution of the mean amplitude of MEPPs does not follow a normal Gaussian distribution suggesting the presence of two clearly distinct neuromuscular junction groups. This fits previous morphological evidence of two populations of motor units in SOD1 mice: one (1) with well-preserved axonal branches sometimes enlarged as the result of re-innervation, and the other (2) with degenerating axon terminals occasionally with no muscular contacts at all [45]. Heterogeneity in the degree of innervation of diaphragm muscle fibers from symptomatic SOD1(G93A) mice has also been reported [46]. This agrees with our functional observations of some symptomatic SOD1(G93A) neuromuscular junctions showing only synchronous and asynchronous spontaneous and no evoked responses. This pattern has been reported to occur in other motor neuron diseases [46] and after axotomy [46], and it is related to deficient innervation. The existence of different neuromuscular junctions within the same muscle is commonly seen in conditions where there is a scattered temporal and spatial pattern of denervation and re-innervation processes across muscle fibers [42,47]. Heterogeneity of muscle fiber function within a motor unit also occurs in ALS patients [48]. Indeed electromyographic recordings show the presence of unstable fasciculation potentials not recruited by voluntary contraction [48]. Worthwhile to note that those unstable fasciculation potentials are most likely to be associated with a re-innervation process and that they occur at an early phase of the disease [48].

Neuromuscular junctions from group SOD1a presented a significant decrease in the mean amplitude of both EPPs and MEPPs and a slower mean rise-time of MEPPs, when compared to adult controls. All these changes suggest a reduction in the influx of cations into the muscle fibers and could be related to (1) a lower sensitivity of the muscle fiber to acetylcholine. This is compatible with previous evidence that neuromuscular junctions from symptomatic SOD1(G93A) EDL muscle display post-synaptic fragmentation and decreased acetylcholine receptors density ( [46]; but see 41), and similarly with what has been reported to occur during aging [49] and in neuromuscular junction disorders such as Duchenne Muscular Dystrophy [50]. However (2), changes in the morphology of diaphragm muscle fibers, as reported in the gastrocnemius from endstage SOD1(G93A) mice [43], or (3) denervation/re-innervation processes which lead to an increase in the distance between the nerve terminal and the motor endplate (and consequently to a reduction in the neuromuscular junction safety factor) cannot be excluded.

## Conclusion

The work herein reported shows that the neuromuscular transmission of SOD1(G93A) mice is enhanced in the pre-symptomatic phase. This probably results from an early maturation mechanism or a compensatory response in order to overcome early denervation and sustain effective contraction. The early changes in the neuromuscular transmission of SOD1(G93A) mice strongly support the idea that ALS associated events start long before symptoms onset. In the symptomatic phase, the detection of mixed populations of neuromuscular junctions agree with the hypothesis that the diaphragm of SOD1(G93A) mice is undergoing cycles of denervation/re-innervation. Results also show that the impairment in the neuromuscular transmission of symptomatic SOD1(G93A) mice are mostly due to postsynaptic changes. Whether these changes at the muscle endplate, which occur at early symptomatic stage, reflect thereafter into nerve inhibition and later in a dying-back neuronal degeneration requires further investigation.
